# Supplementary material for: Improved immunoassay sensitivity and specificity using single-molecule colocalization
Source: Nat Commun. 2022 Sep 12;13:5359. doi: 10.1038/s41467-022-32796-x (PMC9468026; doi:10.1038/s41467-022-32796-x)
Supplement: Supplementary file 1 — Supplementary Information [file 41467_2022_32796_MOESM1_ESM.pdf]

Supplementary information

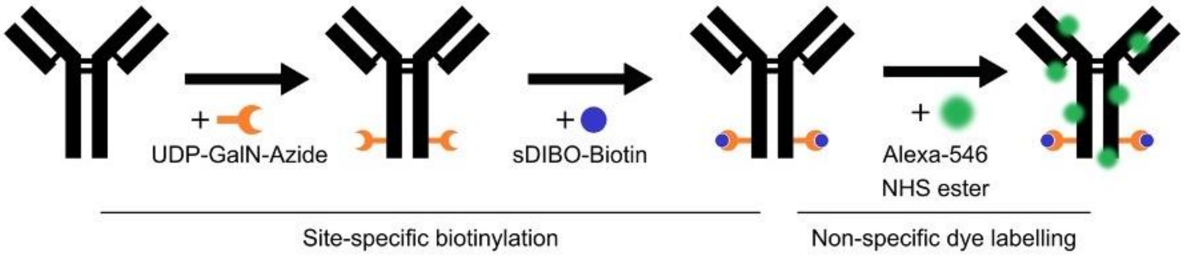

Supplementary Figure 1: Strategy for site-specific and non-specific cAb labeling with fluorophores and biotin labels.

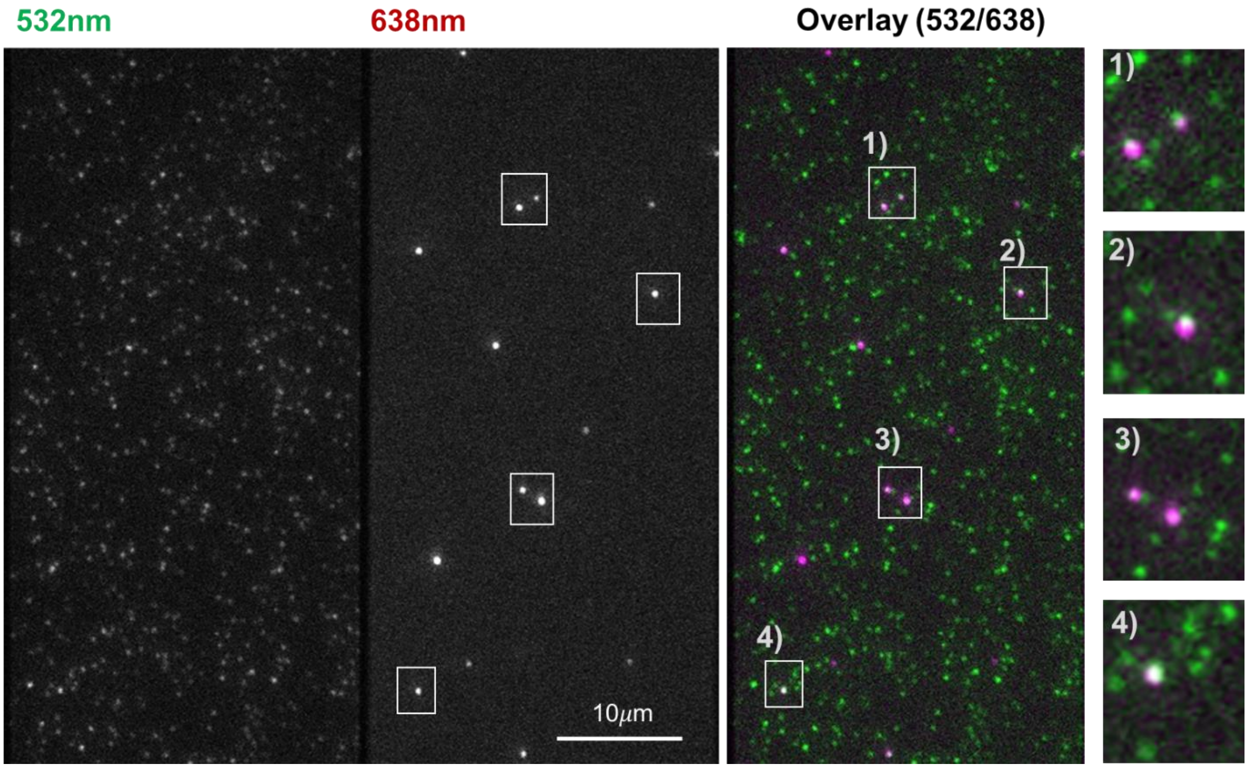

Supplementary Figure 2: Fluorescence images of Alexa 546 (left), Alexa 647 (middle) and the overlay of those two channels (right) showing specific binding at low concentrations (50 nM) of dAb in the presence of 100 pM TNF-α in buffer. Far-right panels show magnification of labeled regions of interest.

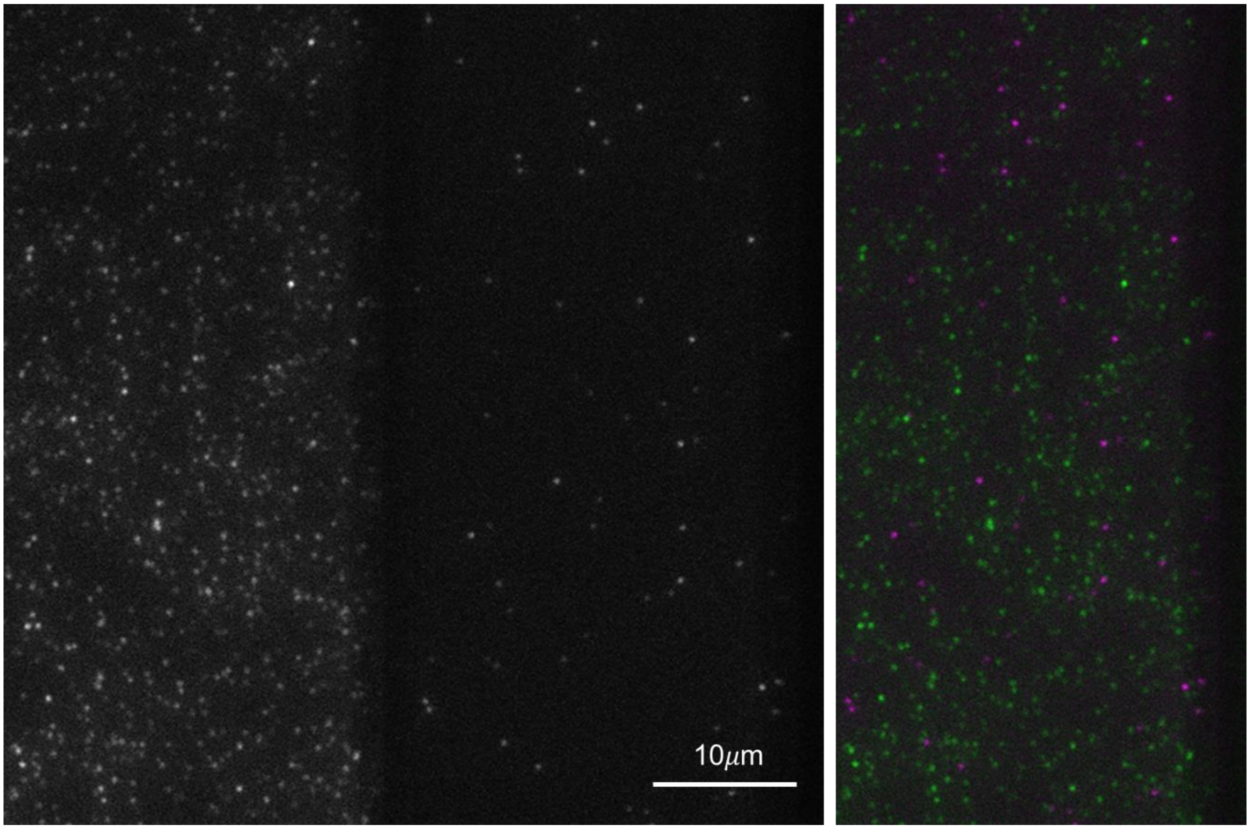

Supplementary Figure 3: Fluorescence images of Alexa 546 (green, left), Alexa 647 (red, middle), and overlays of the two channels (right) showing high levels of non-specific binding at high concentrations (800 nM) of dAb in the absence of TNF-α**.**

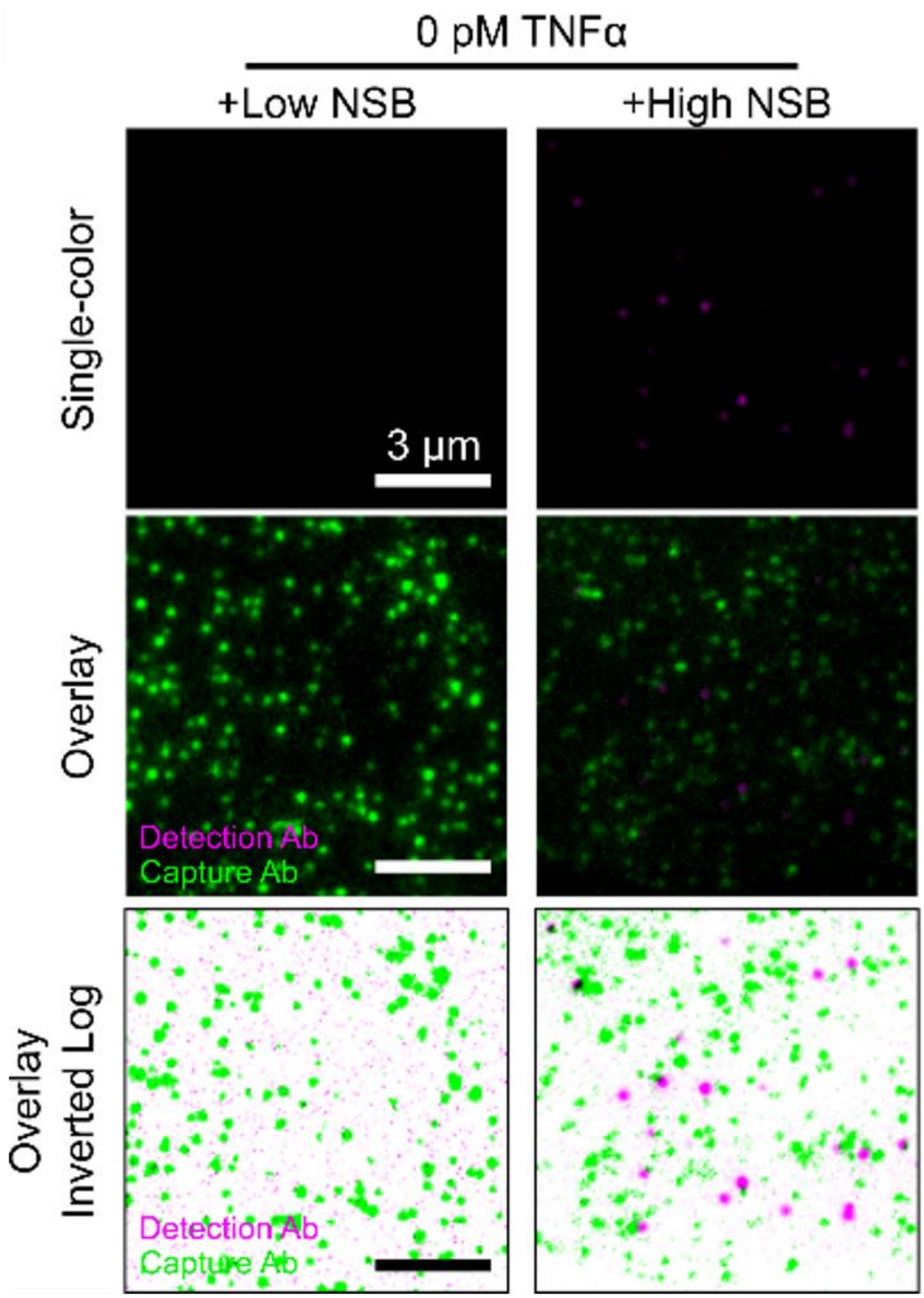

Supplementary Figure 4: Enlarged single-color fluorescence images of dAb only (top), two-color images of cAb and dAb (middle), and log-scale inverted composite images of two-color detection (bottom) in the absence of TNF-α and with 50 nM (left) or 500 nM (right) dAb. Dark spots in the bottom panels represent colocalized signal from the two fluorophores.

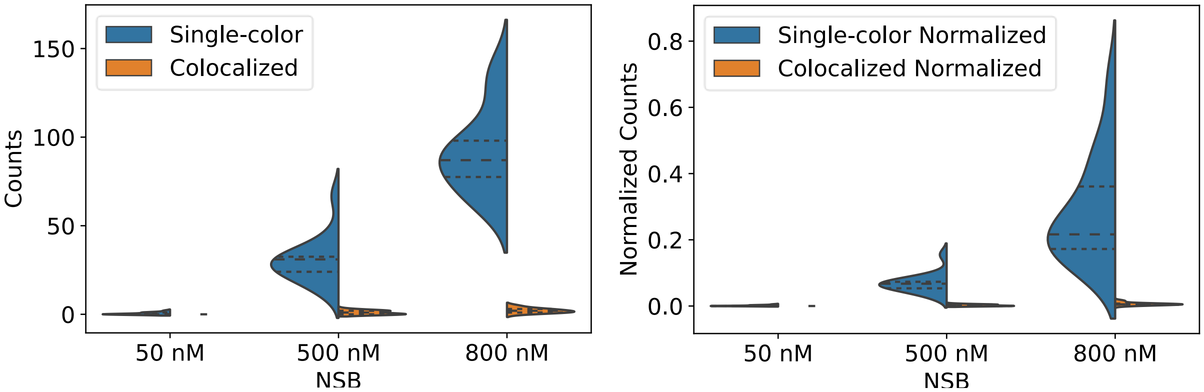

Supplementary Figure 5: Number of counts observed with increasing concentrations of dAb (50, 500, and 800 nM) in the absence of TNF-α using single-color and two-color analysis approaches with absolute counts (left) and cAb-normalized counts (right).

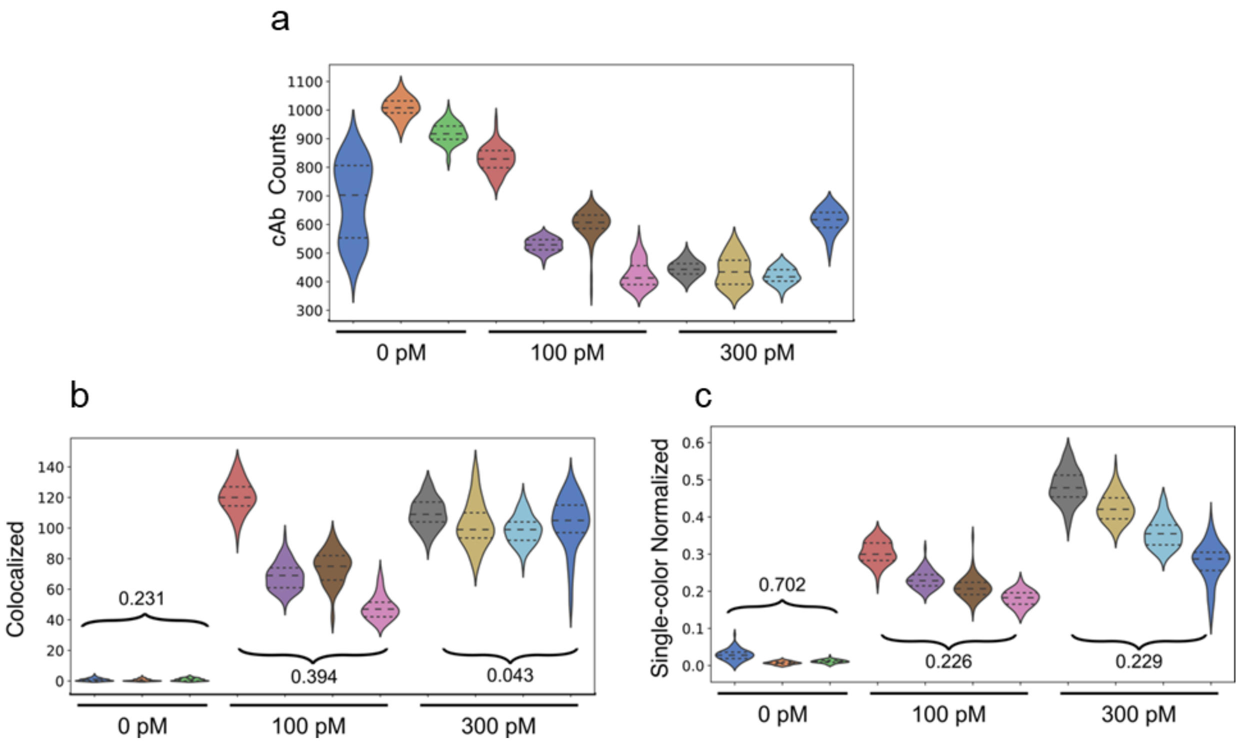

Supplementary Figure 6: a Distribution of absolute cAb counts, **b** colocalized dAb and cAb counts, and **c** single-color normalized dAb counts across 11 coverslips and three different TNF-α concentrations. Numbers in plots B and C present CVs for a given condition.

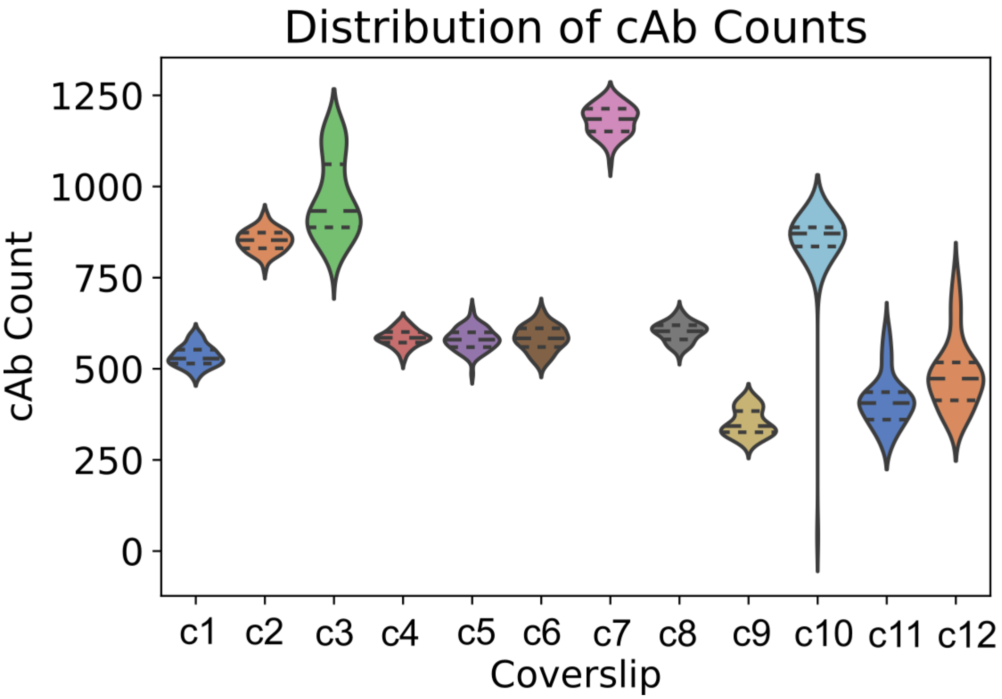

Supplementary Figure 7: Heterogeneity in the distributions of Alexa Fluor 546-labeled cAb. Number of counts across 12 different coverslips (128 different FOVs from each coverslip) after an overnight incubation with target/dAb in buffer.

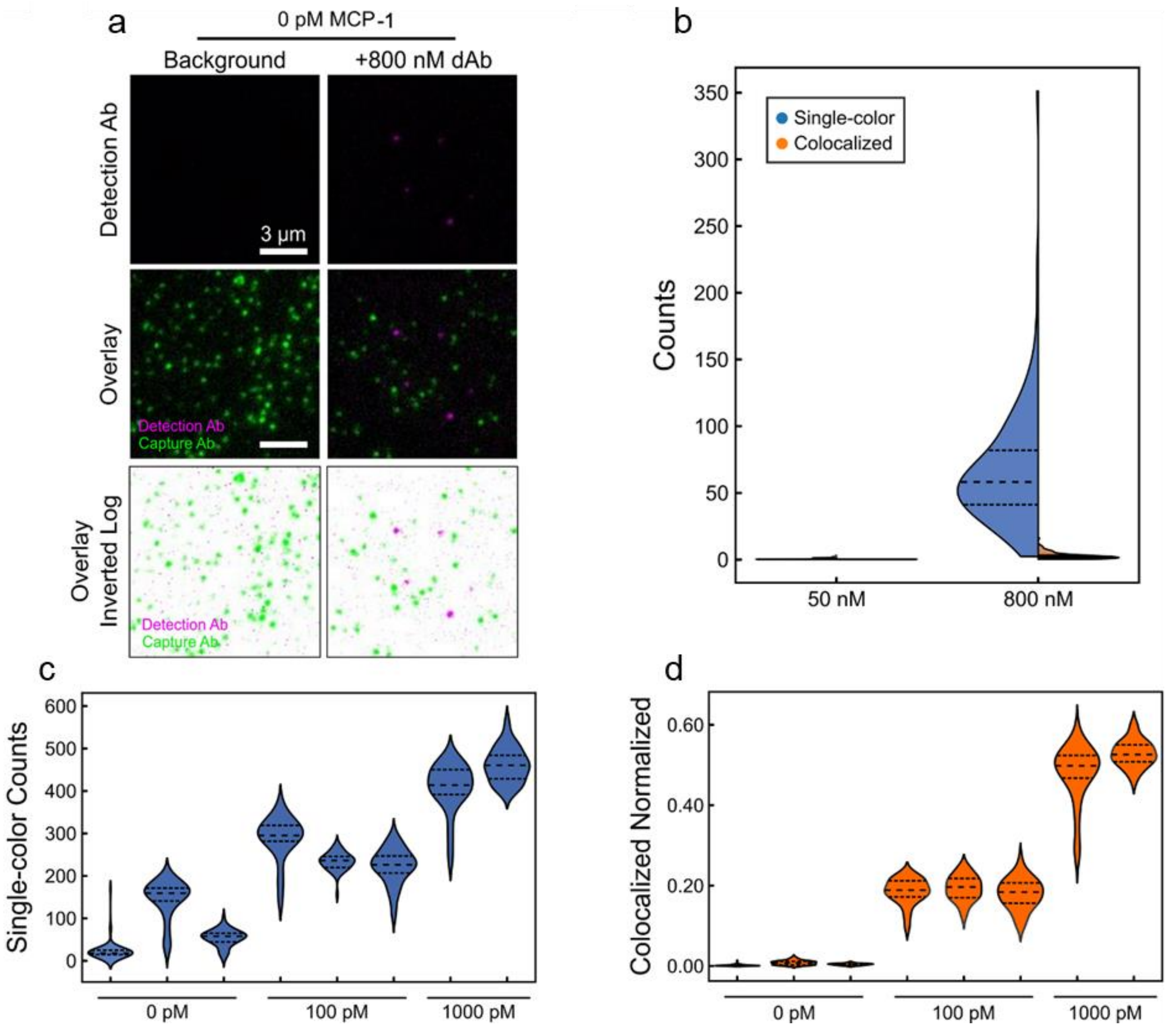

Supplementary Figure 8: Generalizability of SiMCA to a second protein analyte, monocyte chemoattractant protein-1 (MCP-1). Just like TNF- α, we passivated a glass coverslip with a mixture of PEG and PEG-biotin, and then treated with neutravidin and biotinylated, Alexa-546-tagged capture antibodies (cAbs). The surface is then incubated with a solution of the MCP-1 target biomolecule and Alexa-647-labeled detection antibody (dAb) **(See Methods)**. **a** Single-color fluorescence images of dAb only (top), two-color images of cAb and dAb (middle), and log-scale inverted composite images of two-color detection (bottom) in the absence of MCP-1 with 50 nM (left) or 800 nM (right) dAb. Dark spots in the bottom panels represent colocalized signal from the two fluorophores.**b** Distributions of absolute single-color and colocalized counts across 128 fields of view (FOVs). Dashed lines demarcate quartiles of the distribution. **c** Absolute number of dAbs per fields of view. **d** Normalized, colocalized counts across different coverslips and MCP-1 concentrations. Each violin represents 128 FOVs per coverslip (64 per channel).

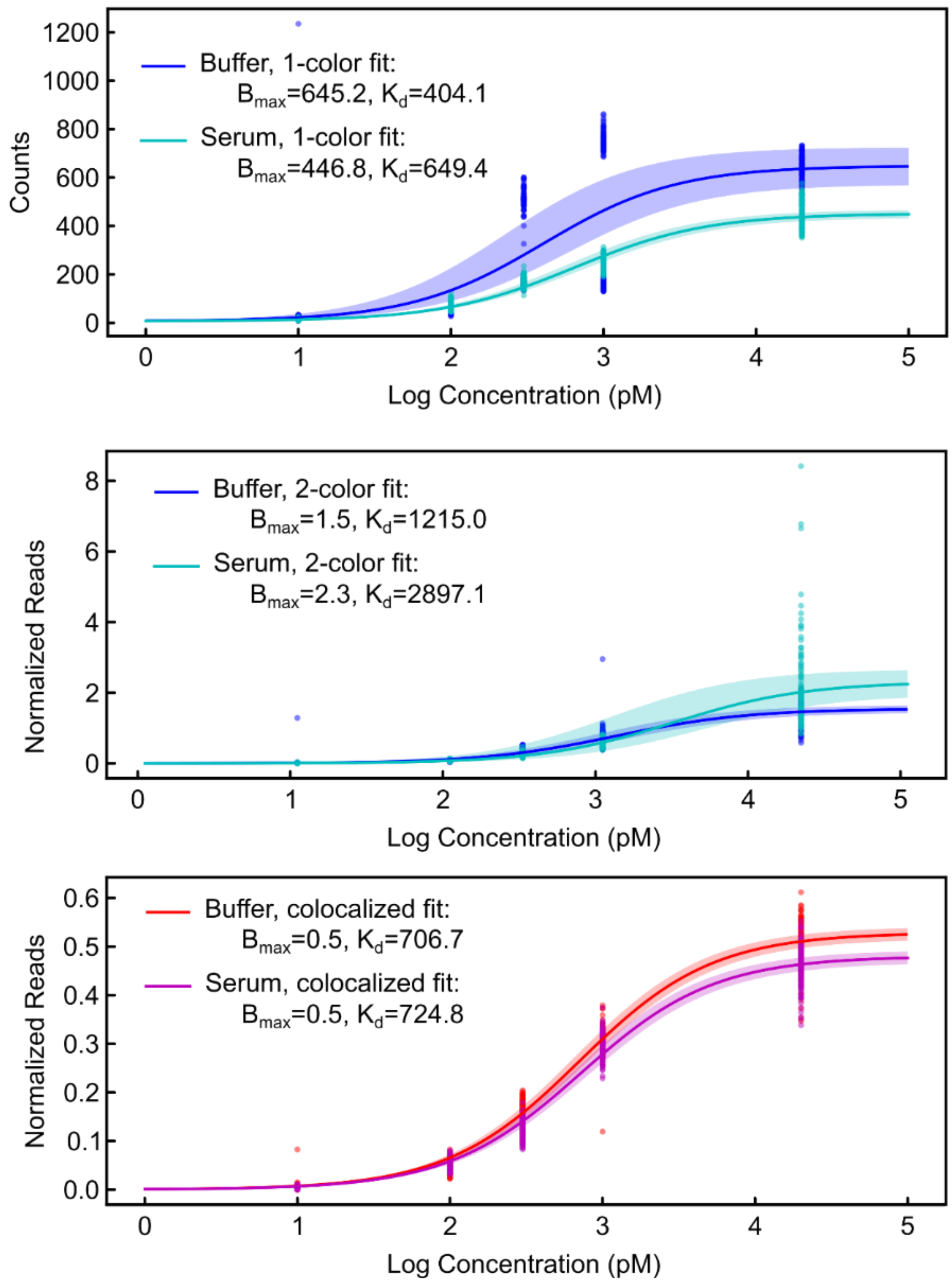

Supplementary Figure 9: Absolute single-color dAb, single-color normalized, and colocalized, normalized counts from each FOV and their respective binding curve fits. 2-sigma confidence curves are shaded.

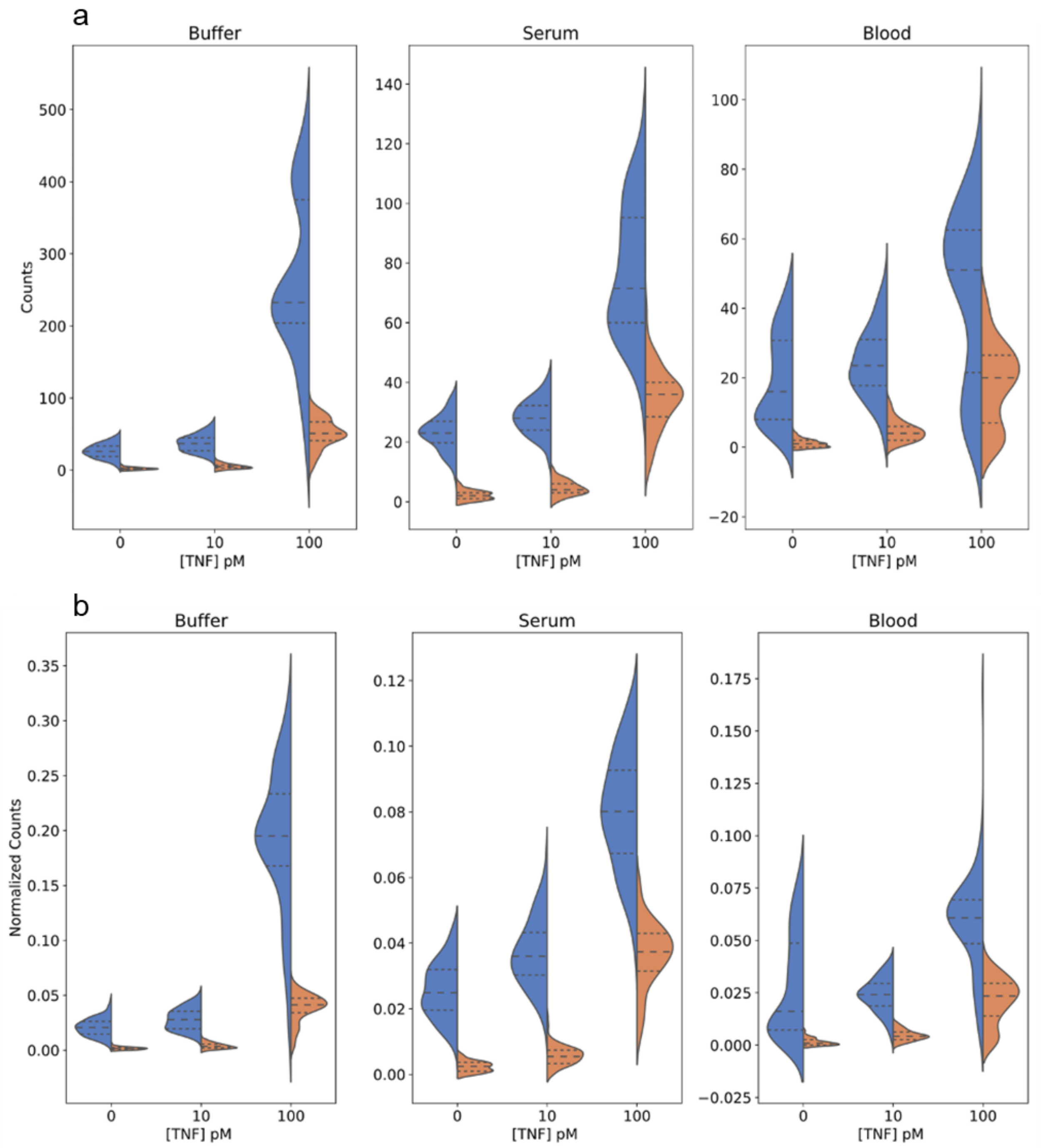

Supplementary Figure 10: Distribution of **a** absolute and **b** normalized single-color dAb (blue) and colocalized counts (orange) from 0, 10, and 100 pM TNF-α in buffer (left), 70% chicken serum (middle), and 70% blood (right). Dashed lines separate quartiles of the distribution.

$$cAb + TNF \underset{K_{D1} \approx 1\ nM}{\leftrightarrow} cAb{\cdot}TNF \underset{K_{D2} \approx 1\ nM}{\leftrightarrow} cAb{\cdot}TNF{\cdot}dAb$$

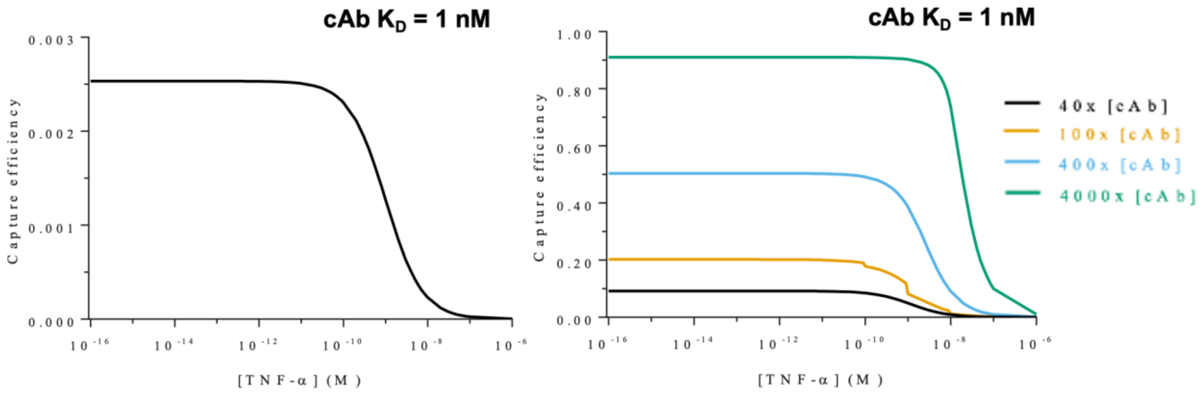

Supplementary Figure 11: Predicted equilibrium binding (capture efficiency) with an estimated effective [cAb] of 2 pM (**left**). Increasing cAb density improves target capture efficiency by more than 100-fold (**right**). Note that these plots assume that all captured TNF- α gets labeled by a dAb, where [dAb] > 50 nM and [TNF-α] ≥ 10 pM, such that we are not limited by depletion effects. This explains why the plots were generated only by using the first equilibrium reaction (see Ref. 25).

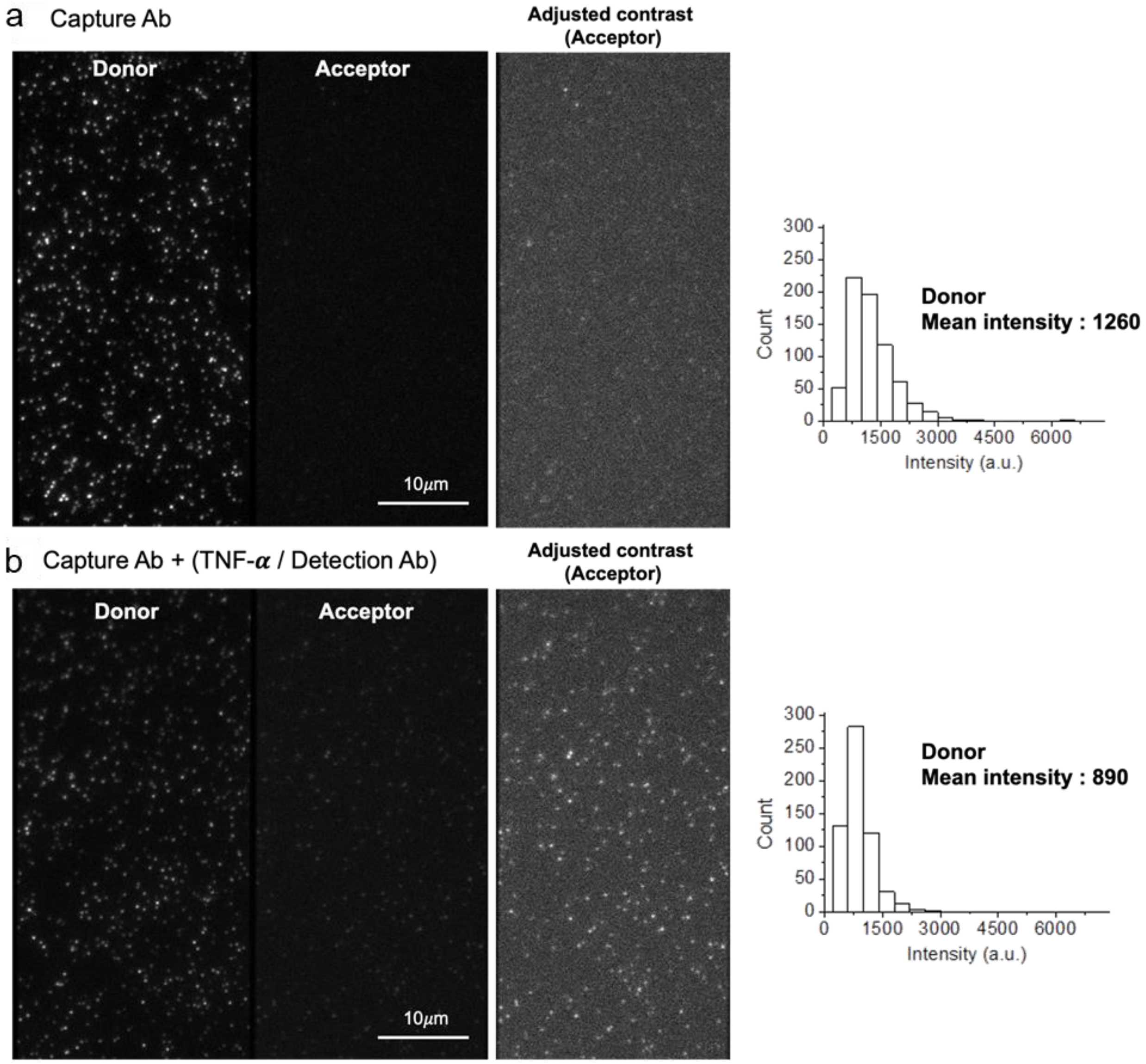

Supplementary Figure 12: Because TNF-α is a small protein, we can detect Förster resonance energy transfer (FRET) between donor fluorophores on the cAb and acceptor fluorophores on the dAb upon binding the protein target. **a** Fluorescence images (left) and donor mean intensities (right) of cAb upon 532 nm excitation before and **b** after target/dAb incubation. The average intensity dropped from 1,260 to 890 after adding target/dAb, and we also observed an increase in the intensity of the acceptor channel upon 532 nm excitation. Adjusted contrast images for the acceptor channel are shown on the right to highlight the intensity increase in the acceptor channel. Scale bar = 10 μm.

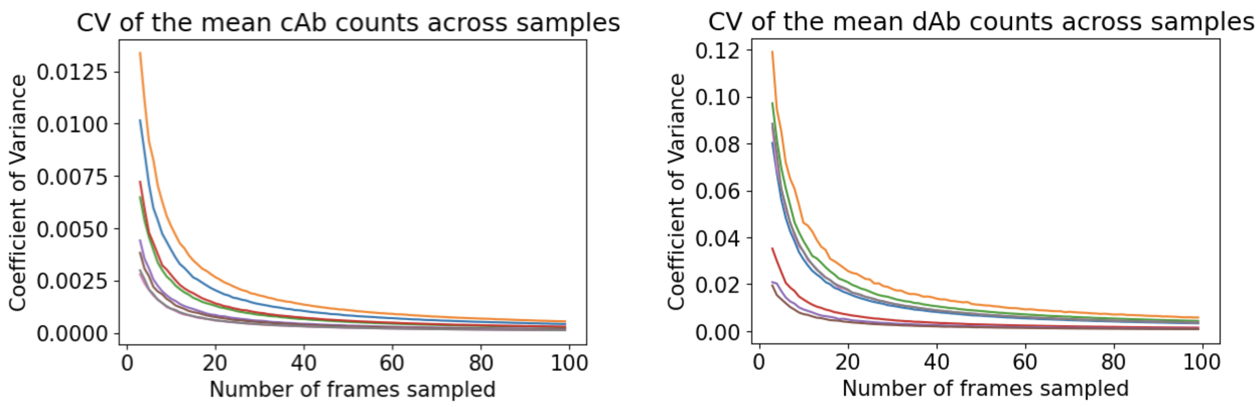

Supplementary Figure 13: Calculated CVs of dAb and cAb counts after sampling 3–100 FOVs for eight coverslips (bootstrapped). A plateau is reached well before the 64 FOV mark.

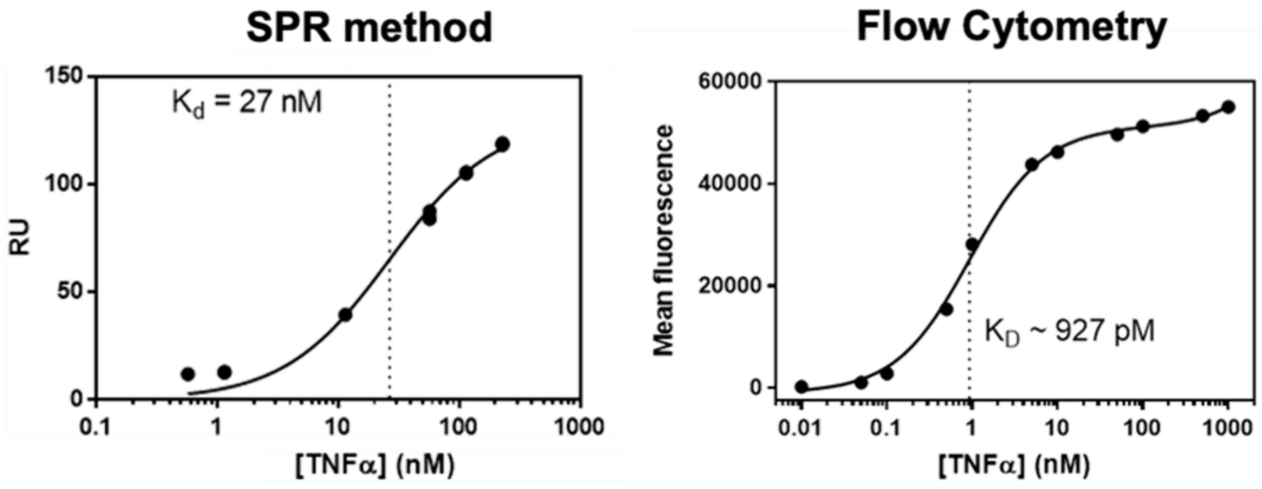

Supplementary Figure 14: Estimated K_d of mAb1 using SPR and flow cytometry techniques. See Reference 37 for more information on mAb11 K_d.

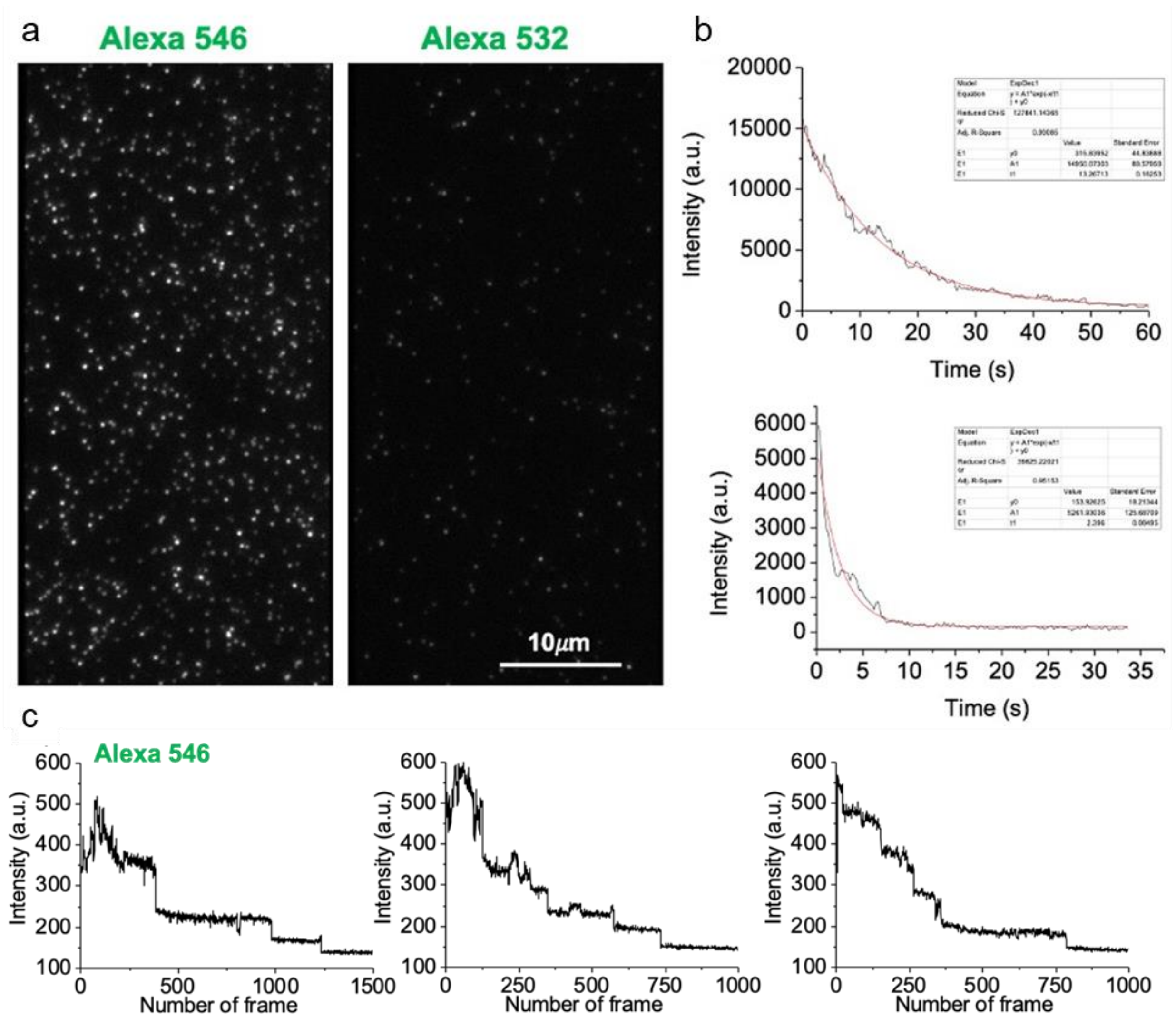

Supplementary Figure 15: Lifetime analysis of Alexa 546- and 532-labeled cAbs with TIRF microscopy. **a** Single image (raw data) of Alexa 546 and Alexa 532 spots on the coverslip surface after excitation at 532 nm for 2 min (at exposure = 200 ms, gain = 3,000, power = 3 mW; see Methods for details). Scale bar = 10 μm. **b** Photobleaching time recorded for Alexa 546 (top) and Alexa 532 (bottom). **c** Examples of the intensity-time trajectories of bleaching of fluorescence emission for Alexa 546-labeled antibodies.

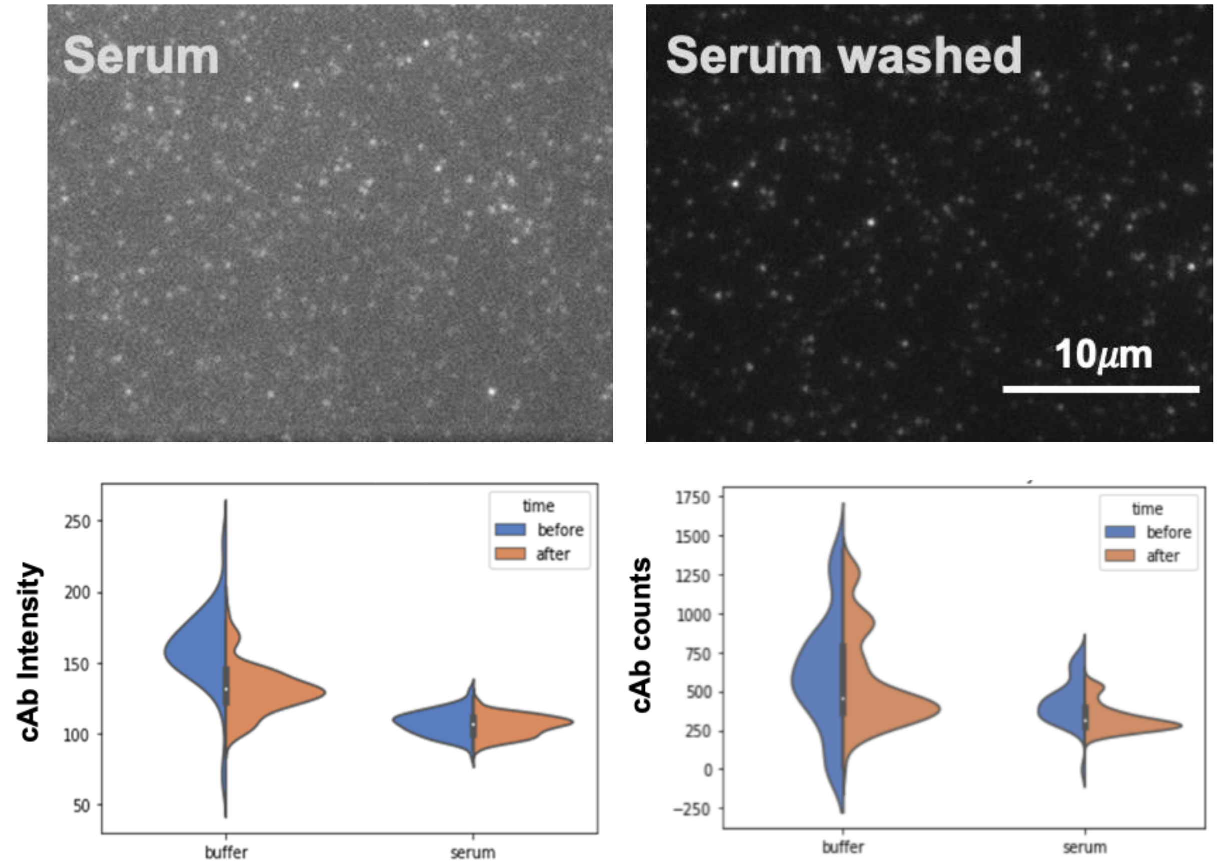

Supplementary Figure 16: cAb fluorescence images in serum before (top left) and after washing with buffer (top right). Distributions of Alexa Fluor 546-labeled cAb intensity (bottom left) and number of counts (bottom right) across six coverslips before and after overnight incubation with buffer and serum. Inner boxplot represent quartiles of distribution. The distribution of cAb intensities and counts remained constant after overnight incubation with buffer and serum, which demonstrates the robustness of the system.

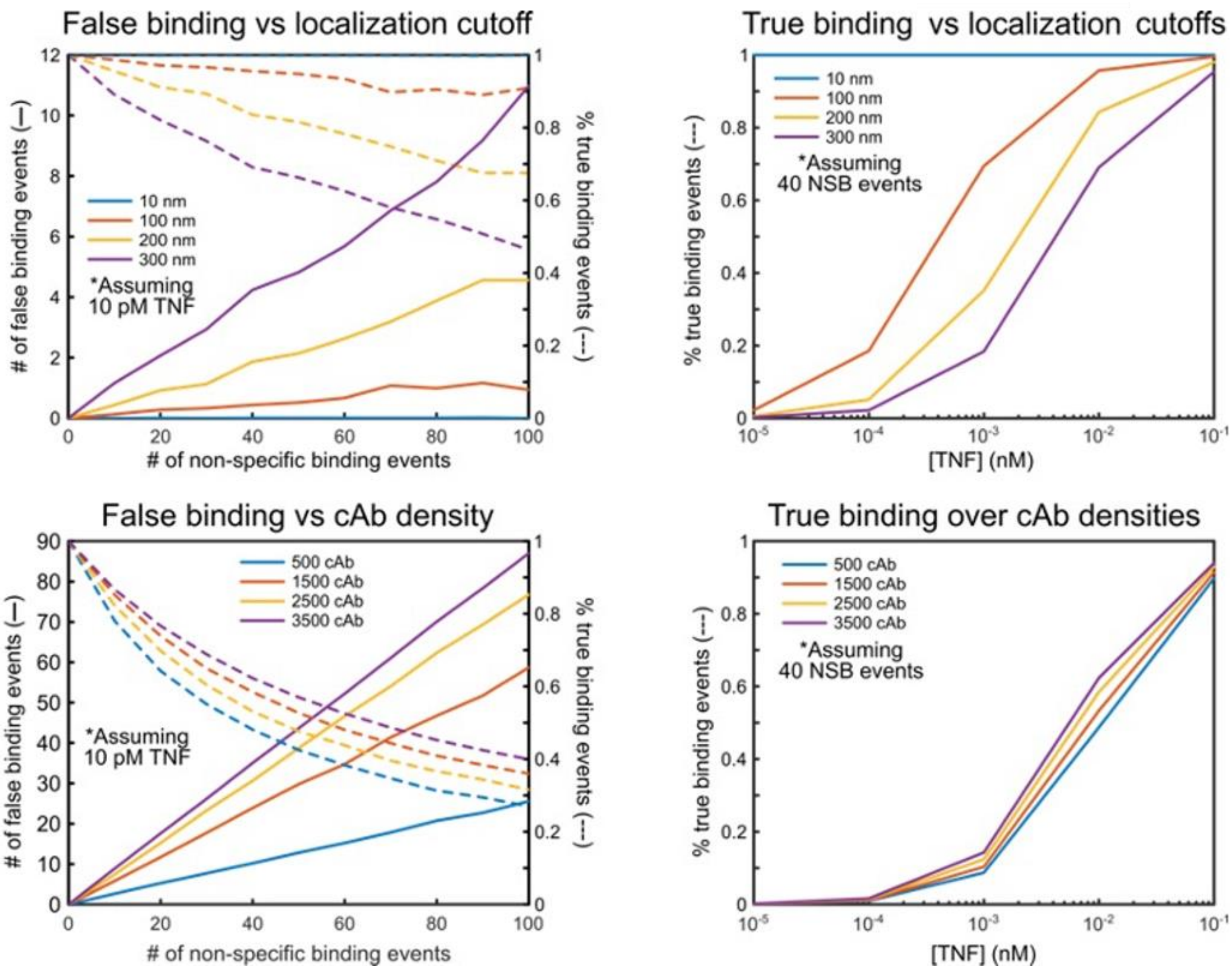

Supplementary Figure 17: Simulations of different colocalization cutoff distances (10–300 nm) to estimate the number of false colocalization events as the number of non-specific binding events increases. Shorter distances (10nm) correspond to a stricter colocalization cutoff which would reduce the number of false colocalizations but requires higher spatial resolution (Top panels). We showed that by increasing the number of capture antibodies on the surface, we would increase the number of false colocalizations but can also lower the limit of detection of the assay (Bottom panels).

Supplementary Table 1: Parameters and respective standard deviation errors from fitting to Langmuir isotherm using single-color and colocalized counts for both normalized and non-normalized methods.

	Buffer			Serum		
	Bmax (CV)	Kd (CV)	LOD (CV)	Bmax (CV)	Kd (CV)	LOD (CV)
1-Color	645 ± 16 (0.024)	404 ± 35 (0.088)	6.6 ± 1.4 (0.210)	446 ± 3.4 (0.008)	649 ± 17 (0.026)	19.4 ± 4 (.212)
Colocalized	235 ± 4.1 (.017)	288 ± 19 (.066)	2.1 ± 0.6 (0.286)	133 ± 2.1 (0.016)	201 ± 12 (.060)	4.9 ± 1.3 (0.256)
1-Color norm	1.55 ± 0.02 (0.014)	1,214 ± 59 (0.049)	11.1 ± 2.2 (0.201)	2.309 ± 0.07 (0.031)	2,897 ± 347 (0.120)	26.4 ± 5.8 (.219)
Colocalized norm	0.528 ± 0.003 (0.005)	706 ± 11.940 (0.017)	3.1 ± 0.9 (0.290)	0.480 ± 0.003 (0.005)	725 ± 13.5 (0.019)	7.7 ± 2.0 (0.258)

Supplementary Table 2: MAPLE Error from bootstrapping using single-color and colocalized counts for normalized and non-normalized methods. CV is displayed in parentheses.

	Buffer	Serum
1-Color	38 ± 1.7 (0.045)	28 ± 2.1 (0.076)
Colocalized	26 ± 2.4 (0.091)	25 ± 1.9 (0.076)
1-Color norm	28 ± 2.0 (0.071)	29 ± 2.2 (0.078)
Colocalized norm	15 ± 2.4 (0.160)	19 ± 2.0 (0.105)

Supplementary Table 3: Poisson distribution of the number of dye labels per antibody.

	Alexa 546 bio-mAb1	Alexa 647-mAb11	Alexa 647 - 10F7	Alexa 546 Bio - 5F3D7
Degree of Labelling	4	3.9	4.4	4.7
Dye per Ab				
0	1.83%	2.02%	1.23%	0.91%
1	7.33%	7.89%	5.40%	4.27%
2	14.65%	15.39%	11.88%	10.05%
3	19.54%	20.01%	17.43%	15.74%
4	19.54%	19.51%	19.17%	18.49%
5	15.63%	15.22%	16.87%	17.38%
6	10.42%	9.89%	12.37%	13.62%
7	5.95%	5.51%	7.78%	9.14%
8	2.98%	2.69%	4.28%	5.37%
9	1.32%	1.16%	2.09%	2.81%
10	0.53%	0.45%	0.92%	1.32%
