# Supplementary material for: Improved immunoassay sensitivity and specificity using single-molecule colocalization
Source: Nat Commun. 2022 Sep 12;13:5359. doi: 10.1038/s41467-022-32796-x (PMC9468026; doi:10.1038/s41467-022-32796-x)
Supplement: Supplementary file 3 — Reporting Summary [file 41467_2022_32796_MOESM3_ESM.pdf]

Corresponding author(s): H. Tom Soh and Alexander Dunn

Last updated by author(s): Jul 7, 2022

Reporting Summary

Nature Portfolio wishes to improve the reproducibility of the work that we publish. This form provides structure for consistency and transparency in reporting. For further information on Nature Portfolio policies, see our Editorial Policies and the Editorial Policy Checklist.

Statistics

For all statistical analyses, confirm that the following items are present in the figure legend, table legend, main text, or Methods section.

n/a	Confirmed	
☐	☒	The exact sample size (n) for each experimental group/condition, given as a discrete number and unit of measurement
☐	☒	A statement on whether measurements were taken from distinct samples or whether the same sample was measured repeatedly
☐	☒	The statistical test(s) used AND whether they are one- or two-sided<br>*Only common tests should be described solely by name; describe more complex techniques in the Methods section.*
☐	☒	A description of all covariates tested
☐	☒	A description of any assumptions or corrections, such as tests of normality and adjustment for multiple comparisons
☐	☒	A full description of the statistical parameters including central tendency (e.g. means) or other basic estimates (e.g. regression coefficient) AND variation (e.g. standard deviation) or associated estimates of uncertainty (e.g. confidence intervals)
☒	☐	For null hypothesis testing, the test statistic (e.g. F, t, r) with confidence intervals, effect sizes, degrees of freedom and P value noted<br>*Give P values as exact values whenever suitable.*
☒	☐	For Bayesian analysis, information on the choice of priors and Markov chain Monte Carlo settings
☒	☐	For hierarchical and complex designs, identification of the appropriate level for tests and full reporting of outcomes
☒	☐	Estimates of effect sizes (e.g. Cohen's d, Pearson's r), indicating how they were calculated

Our web collection on statistics for biologists contains articles on many of the points above.

Software and code

Policy information about availability of computer code

Data collection	Micro-Manager version 2.0.0 was used to operate and collect data from the Nikon TiE.
Data analysis	Custom code with demonstration files available here: https://github.com/newmanst/simca-pub.<br>Figure reproduction instructions and respective jupyter notebooks can be pushed to github upon request.

For manuscripts utilizing custom algorithms or software that are central to the research but not yet described in published literature, software must be made available to editors and reviewers. We strongly encourage code deposition in a community repository (e.g. GitHub). See the Nature Portfolio guidelines for submitting code & software for further information.

Data

Policy information about availability of data

All manuscripts must include a data availability statement. This statement should provide the following information, where applicable:

- Accession codes, unique identifiers, or web links for publicly available datasets
- A description of any restrictions on data availability
- For clinical datasets or third party data, please ensure that the statement adheres to our policy

The data generated in this work are available in the Stanford Digital Repository under accession code https://purl.stanford.edu/bc494tq1762. A subset of data for demonstration purposes are available at https://github.com/newmanst/simca-pub.

Field-specific reporting

Please select the one below that is the best fit for your research. If you are not sure, read the appropriate sections before making your selection.

- [x] Life sciences
- [ ] Behavioural & social sciences
- [ ] Ecological, evolutionary & environmental sciences

For a reference copy of the document with all sections, see nature.com/documents/nr-reporting-summary-flat.pdf

Life sciences study design

All studies must disclose on these points even when the disclosure is negative.

Sample size	We determined 16 frame of views (FOVs) provided minimum sufficiency for each channel/coverslip by analyzing bootstrapped variance of a range of FOVs from 3 coverslips from our initial experiment. We calculated that variance of the mean spot counts across FOVs dropped significantly beyond using 16 FOVs, and consistently plateaued before 64 FOVs. We chose 64 as a safe cut off much beyond the inflection point as shown in Supplementary Figure 13.
Data exclusions	Coverslips were excluded from analysis when impurities/contamination were present in the sample, which was indicated by the presence of micron size bright fluorescent spots covering large areas of coverslips surface. This could be due to buffer or Ab sample contamination. FOVs were removed due to bubbles or large dust particles being introduced at the FOV. Since the dynamics of target and dAb binding to the coverslip surface could be significantly altered in these frames, we removed these in an automated post-imaging step described in the Image segmentation and registration section in the methods.
Replication	A minimum of 2 coverslips were prepared, imaged and analyzed for each condition. A total of 16 or 64, fields of view (FOVs) were acquired and analyzed for each coverslip. More replicates were performed for the study, but due to laser damage/change and microscope/camera re-alignment issues, they were dismissed from the study for consistency. All duplicates presented in this work were done using the same laser/power, exposure time / Gain and same microscope/camera alignment. All attempt at replications were successful.
Randomization	Coverslips were randomly allocated for TNF-alpha/dAb and MCP-1/dAb samples of different concentrations.
Blinding	Data collection: Blinded. Samples were prepared in the Soh lab (numbered, and each number was associated to a concentration written in notebook in Soh lab) and imaged afterwards in Dunn lab. Concentrations were revealed after imaging.<br>Data analysis: Blinded. 64 FOV per coverslip were analyzed automatically using custom image analysis software (provided on github) without bias from investigator. Only after spot counting was done, were concentrations revealed.

Reporting for specific materials, systems and methods

We require information from authors about some types of materials, experimental systems and methods used in many studies. Here, indicate whether each material, system or method listed is relevant to your study. If you are not sure if a list item applies to your research, read the appropriate section before selecting a response.

Materials & experimental systems

n/a	Involved in the study	
☐	☒	Antibodies
☒	☐	Eukaryotic cell lines
☒	☐	Palaeontology and archaeology
☒	☐	Animals and other organisms
☐	☒	Human research participants
☒	☐	Clinical data
☒	☐	Dual use research of concern

Methods

n/a	Involved in the study	
☒	☐	ChIP-seq
☒	☐	Flow cytometry
☒	☐	MRI-based neuroimaging

Antibodies

Antibodies used	Anti-human TNF-α cAb (mAb1, BioLegend, Catalog #502802) and Alexa Fluor® 647 Anti-human TNF-α dAbs (mAb11, BioLegend, Catalog # 502916) , Mouse anti-human MCP-1 antibody (clone 5D3-F7; #BD551226, BD Biosciences), Mouse anti-human MCP-1 antibody (clone 10F7; #BD555055, BD Biosciences)

Validation	Technical data-sheet for mAb1: https://www.biolegend.com/en-us/search-results/purified-anti-human-tnf-alpha-antibody-1010<br>Technical data-sheet for Alexa-647 mAb11: https://www.biolegend.com/en-us/products/alexa-fluor-647-anti-human-tnf-alpha-antibody-2751?GroupID=GROUP24 .<br>Technical data-sheet for Mouse anti-human MCP-1 antibody (clone 10F7; #BDB555055, BD Biosciences): https://www.bdbiosciences.com/content/bdb/paths/generate-tds-document.us.555055.pdf<br>Technical data-sheet for Mouse anti-human MCP-1 antibody (clone 5D3-F7; #BD551226, BD Biosciences): https://www.bdbiosciences.com/content/bdb/paths/generate-tds-document.us.551226.pdf

Human research participants

Policy information about studies involving human research participants

Population characteristics	N/A. Healthy human blood purchased from BioIVT.
Recruitment	No recruitment involved.
Ethics oversight	Following the safety protocols of Stanford University, there is no specific approval for human blood or other fluids that we have purchase or used since they are not infectious and were pre-screened and free of viruses and pathogens but are always handled as if they could be infectious. i.e. using universal precautions. APB approval is required for products designated BSLII and up.

Note that full information on the approval of the study protocol must also be provided in the manuscript.
